# Dynamics of the fecal microbiome in patients with recurrent and nonrecurrent *Clostridium difficile* infection

**DOI:** 10.1186/s13073-016-0298-8

**Published:** 2016-04-27

**Authors:** Anna Maria Seekatz, Krishna Rao, Kavitha Santhosh, Vincent Bensan Young

**Affiliations:** Department of Internal Medicine/Division of Infectious Disease, University of Michigan Medical School, Ann Arbor, MI USA; Department of Microbiology & Immunology, University of Michigan Medical School, Ann Arbor, MI 48109 USA; Ann Arbor Veterans Affairs Healthcare System, Ann Arbor, MI USA

## Abstract

**Background:**

Recurrent *Clostridium difficile* infection (CDI) remains problematic, with up to 30 % of individuals diagnosed with primary CDI experiencing at least one episode of recurrence. The success of microbial-based therapeutics, such as fecal microbiota transplantation, for the treatment of recurrent CDI underscores the importance of restoring the microbiota. However, few studies have looked at the microbial factors that contribute to the development of recurrent disease. Here we compare microbial changes over time in patients with or without recurrence to identify microbial signatures associated with the development of recurrence.

**Methods:**

We used 16S rRNA-encoding gene sequence analysis to compare the fecal microbiota of 93 patients with recurrent and nonrecurrent CDI, sampled longitudinally. Cross-group and intra-individual differences in microbial community diversity and similarity were compared prior to the development of recurrent disease and over time.

**Results:**

Samples from these patient groups exhibited variable community profiles, clustering into four distinct community groups. Cross-group comparison of the index sample collected from patients that did or did not develop recurrence revealed differences in diversity and community structure (analysis of molecular variance, *p* < 0.05). Intra-individual comparisons of the microbiota were more informative and samples from recurrent patients were less likely to recover in diversity (Chi-square test, *p* < 0.005), exhibiting less community similarity overall (Kruskal–Wallis test, *p* < 0.05). Interestingly, patients with severe disease harbored a significantly less diverse community, a trend that was observed across both nonrecurrent and recurrent patient groups (Wilcoxon test, *p* < 0.05).

**Conclusions:**

To date, this study represents one of the largest studies focused on the relationship between predictive signals from the gut microbiota and the development of recurrent CDI. Our data demonstrate that specific microbiota-derived characteristics associate with disease severity and recurrence and that future studies could incorporate these characteristics into predictive models.

**Electronic supplementary material:**

The online version of this article (doi:10.1186/s13073-016-0298-8) contains supplementary material, which is available to authorized users.

## Background

*Clostridium difficile* infection (CDI) has become one of the most prevalent hospital-acquired infections in recent years [1]. Adding to the impact of CDI, recurrent disease affects 20–30 % of patients after an initial diagnosis [2]. Although multiple factors are associated with recurrence, the exact contributions of these factors to the development of recurrence in certain patients remain undetermined. Strains of *C. difficile* belonging to the 027 ribotype have been associated with higher rates of recurrence at some institutions [3]. Similarly, certain antibiotic treatment options have also been associated with more recurrence [4]. Failure to develop an adaptive immune response against the *C. difficile* toxins has also been associated with increased risk for recurrence [5]. Since the relationship between the gastrointestinal microbiota, i.e., the indigenous microbes of the gastrointestinal tract, and the development of CDI has been well-established, specific differences in the gastrointestinal microbiota may contribute to susceptibility to recurrence.

The importance of the gut microbiota in recovering from a recurrent CDI cycle has been previously demonstrated. Fecal microbiota transplantation (FMT) is one of the most effective therapies for recurrent CDI, with an over 90 % success rate [6–8]. Several studies have observed a significant recovery in the diversity of the microbial community following FMT, although the specific microbes that contribute to recovery are variable between patients [9–11]. However, studies have not followed CDI patients over time to compare those that do or do not develop recurrent disease.

The objective of this study was to compare the gastrointestinal microbiota of patients diagnosed with CDI, with or without recurrence. We examined the microbiota at initial diagnosis (index) time points, as well as the longitudinal changes in the microbiota of patients over time. We observed marked differences in the recovery of recurrent patients compared with nonrecurrent cases. This study represents the most comprehensive exploration of the microbiota during development of recurrent CDI.

## Methods

### Study design, patient population, and sample collection

**Fig. 1:**
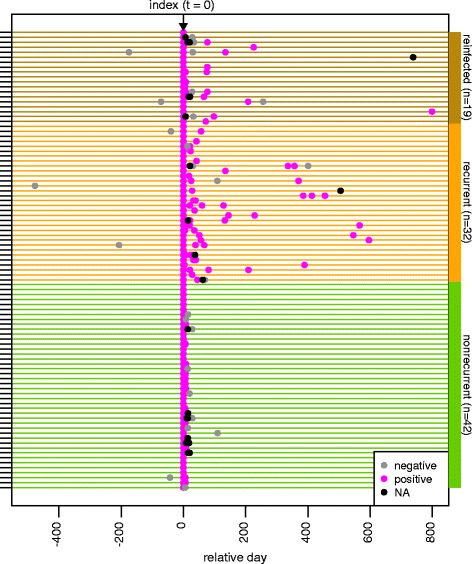
Study design and sample collection timeline. Relative timeline (days) of samples collected from patients diagnosed with initial *Clostridium difficile* infection (CDI) (index sample = 0 days) categorized into three patient groups (*nonrecurrent*, *recurrent*, and *reinfected*). Patients who did not develop recurrence (*n* = 42) remained free of a subsequent CDI diagnosis. Patients with recurrent disease (*n* = 32) were diagnosed with CDI (positive clinical lab result) 14–56 days following index sample collection. Patients diagnosed with another CDI index outside of the recurrence window (>56 days) were considered reinfected (*n* = 19) NA = test not available

Fecal samples for this study were selected retrospectively from a biorepository created as part of the NIH Enterics Research Investigational Network (ERIN) study, and encompassed patients who received care at the University of Michigan Health System (UMHS) from October 2010 to June 2014. The ERIN study obtained the index fecal specimens through collection of discarded fecal material after *C. difficile* testing by the clinical microbiology laboratory and follow-up specimens through informed consent of patients who were aged over 18 years and not pregnant. Patients included in this study were selected based on the availability of multiple fecal samples following an initial CDI diagnosis (Fig. 1). The number of longitudinal samples varied between patients, as did the interval between individual samples (Table 1; Additional file 1: Table S1). Clinical data were extracted from the electronic medical record through both automated query and manual chart review by infectious disease clinicians (KR and DM) (Additional file 1: Table S1).

**Table 1:** Patient and sample metadata

Patient group	Number of patients (total *n*, %)	Number of samples (total *n*, mean *n*/patient, range)	Relative distance to index sampling (median days, range)	Age (years; mean, range)	Sex (*n*, %)	Number of patients with known IBD (total *n*, %)	Number of patients with known antibiotic exposure prior to CDI (total *n*, %)
Nonrecurrent	42 (66.3 %)	80, 2/patient (1–4 samples)	8 (1–110)	57.7 (18–89)	F: 18 (43 %) M: 24 (57 %)	2 (5 %)	15 (36 %)
Recurrent	32 (19.4 %)	94, 3/patient (1–6 samples)	42 (3–596)	53.9 (26–84)	F: 22 (69 %) M: 10 (31 %)	0	5 (15 %)
Reinfected	19 (14.3 %)	51, 3/patient (1–7 samples)	134 (5–799)	56.4 (23–78)	F: 10 (53 %) M: 9 (47 %)	1 (5 %)	5 (26 %)
Total	93	225, 2.4/patient (1–7 samples)	31 (1–799)	55.6 (18–89)	F: 50 (54 %) M: 43 (46 %)	3 (3 %)	25 (27 %)

The number of patients and samples per group, with age, sex, and relative distance to initial sampling per patient group. *F* female, *M* male

Samples were collected in Cary-Blair transport media as per hospital protocol during the study period. Clinical testing for CDI was performed at the discretion of the patients’ inpatient care team, following institutional and national guidelines that recommend testing of only symptomatic patients [12]. Testing for CDI in the clinical lab followed a two-stage algorithm employing the *C. diff* Quik Chek Complete (TechLab, Blacksburg, Virginia, USA), which detects glutamate dehydrogenase (GDH) antigen and toxins A and B via enzyme immunoassay, with confirmation by polymerase chain reaction (PCR) for the *C. difficile* toxin gene, *tcdB*, if the toxin and GDH results from the Quik Chek test were discordant. The *C. difficile* clinical status (“positive” or “negative”) used in this study is based on the presence of a positive test and symptoms and is listed under “clinical lab result” in Additional file 1: Table S1. Index, recurrent, and reinfected cases were defined in conjunction with a positive Quik Chek result with a chart review confirming symptoms of CDI and the patient’s medical history. The “sample status” definition used in Additional file 1: Table S1 and Fig. 6 is based on the Infectious Diseases Society of America (IDSA) surveillance definitions [13] applied to each patient, resulting in categorization of the patient into nonrecurrent, recurrent, or reinfected clinical groups as follows: index (first positive sample collected in study), recurrence (subsequent positive sample 15–56 days from a previous positive sample), reinfection (subsequent positive sample >56 days from a previous positive sample), treatment (sample collected within 14 days of a positive sample, during antibiotic treatment), and recovery (nonrecurrent, non-reinfected sample collected >14 days from a positive sample). Disease severity (based on the IDSA criteria of a circulating white blood cell count >15,000 cells/mm^3^ and/or a serum creatinine >1.5 times the baseline value) [12] was recorded when available (Table 1; Additional file 1: Table S1). Following routine testing in the clinical laboratory, excess sample was transported to the research laboratory and stored at −80 °C before further processing.

We isolated *C. difficile* from each sample as described previously [14, 15]. Fecal samples were grown in taurocholate cycloserine cefoxitin fructose agar (TCCFA) media anaerobically overnight to enrich for *C. difficile* spores, then plated on TCCFA media to isolate single *C. difficile* colonies. The ribotypes of single *C. difficile* isolates were obtained using a high-throughput ribotyping protocol, previously validated at multiple centers [14]. Results from these analyses are listed in Additional file 1: Table S1 under “plating results” and “ribotype”.

### DNA extraction and 16S rRNA sequencing

Total fecal DNA was extracted from 200–300 μl fecal content using the MoBio PowerMag soil isolation kit optimized for the epMotion 5075 TMX (MoBio Laboratories, #271004EP; Eppendorf) using the manufacturer’s instructions, which includes a mechanical bead-beating step. The University of Michigan Host Microbiome core prepped DNA libraries as previously described [16]. In brief, amplification of the 16S V4 region was accomplished using specific barcoded dual index primers as described in Kozich et al. [17]. The PCR reaction included the following: 5 μl of 4 μM stock combined primer set, 0.15 μl of Accuprime High-Fidelity Taq with 2 μl of 10× Accuprime PCR II buffer (Life Technologies, #12346094), 11.85 μl of PCR-grade water, and 1 μl of template. The PCR cycling conditions were as follows: 95 °C for 2 minutes, 30 cycles of 95 °C for 20 s, 55 °C for 15 s, and 72 °C for 5 minutes, and 10 minutes at 72 °C. The DNA library plates were normalized with a SequelPrep normalization kit (Life Technologies, #A10510-01) and pooled. The pooled concentration was quantified using the Kapa Biosystems Library Quantification kit for Illumina platforms (KapaBiosystems, #KK4854) and amplicon size was determined using the Agilent Bioanalyzer high-sensitivity DNA analysis kit (#5067-4626). The MiSeq Reagent 222 kit V2 (#MS-102-2003) was used to sequence the amplicons (500 total cycles) with modifications for the primer set. Illumina’s protocol for library preparation was used for 2 nM libraries, with a final loading concentration of 4 pM spiked with 10 % PhiX for diversity. Paired-end reads of FASTQ files for all samples are available in the Sequence Read Archive under BioProject PRJNA307992 (SRP068473).

### Data processing and analysis

Detailed commands for data processing, presentation, and statistical analysis are available at https://github.com/aseekatz/ERIN.recurrence. Raw sequence files were processed using mothur v1.34.4 [18]. Sequences were trimmed, aligned, and binned, using UCHIME to remove chimeric sequences [19]. A mothur-adapted version of the SILVA rRNA database project (release v119) was used to align the V4 region [20]. Samples with less than 1400 reads were discarded. Sequences were taxonomically classified at 80 % bootstrap minimum using the Wang method to the mothur-adapted RDP database (v10) [21]. Standard and loadable R packages (R Foundation for Statistical Computing, Vienna, Austria, v3.1.0) were used to process the data following processing in mothur. The Partitioning Around Medoids (PAM) clustering algorithm was used to cluster samples into community clusters based on the Jensen–Shannon divergence from phylotype relative abundance in R as conducted previously [22] using the silhouette score to determine the optimal number of clusters (*S(i)* = 0.26, four clusters). A 97 % cutoff was used to bin sequences into operational taxonomic units (OTUs) in mothur for downstream analyses. The inverse Simpson index (*λ*), the Yue and Clayton dissimilarity index (*θ*_*YC*_) [23], and principal coordinates analysis (PCoA) of *θ*_*YC*_ distance were calculated in mothur using OTU abundance. Results were plotted using R. A heatmap of the relative abundance of dominant OTUs was visualized using the R package gplots [24]. Standard R commands were used to visualize the results from linear discriminant analysis (LDA) effect size (LEfSe) in nonrecurrent/recurrent patients or clinically negative/positive samples [25].

### Statistical analysis

Wilcoxon rank sum tests were used to determine significance of binary group comparisons for diversity *λ* and community dissimilarity *θ*_*YC*_. The Kruskal–Wallis was used for comparison of three or more groups. Analysis of molecular variance (AMOVA) was used to compare group communities, as directed in mothur [26]. To compare diversity *λ* between nonrecurrent and recurrent patients, a generalized estimating equation (GEE) model was applied [27]. The R package “geepack” was used to calculate the model, using a first order autoregressive correlation structure and a binomial link-logit family specification [28]. A Chi-squared test was performed to determine distribution of sample categories in the four identified microbial community type clusters and for inference about the variable coefficients’ point estimates from GEE.

## Results

### Distinct microbial community features are present in the feces of patients with severe *C. difficile* infection but are not with recurrent infection

Following an initial diagnosis of CDI (index sample), longitudinal fecal samples were collected from patients with nonrecurrent and recurrent disease and patients who were reinfected with *C. difficile* past the 56-day window of the recurrence definition, as described in “Methods” (Fig. 1, Table 1). The fecal microbiota from each sample was examined by 16S rRNA-encoding gene sequence analysis.

**Fig. 2:**
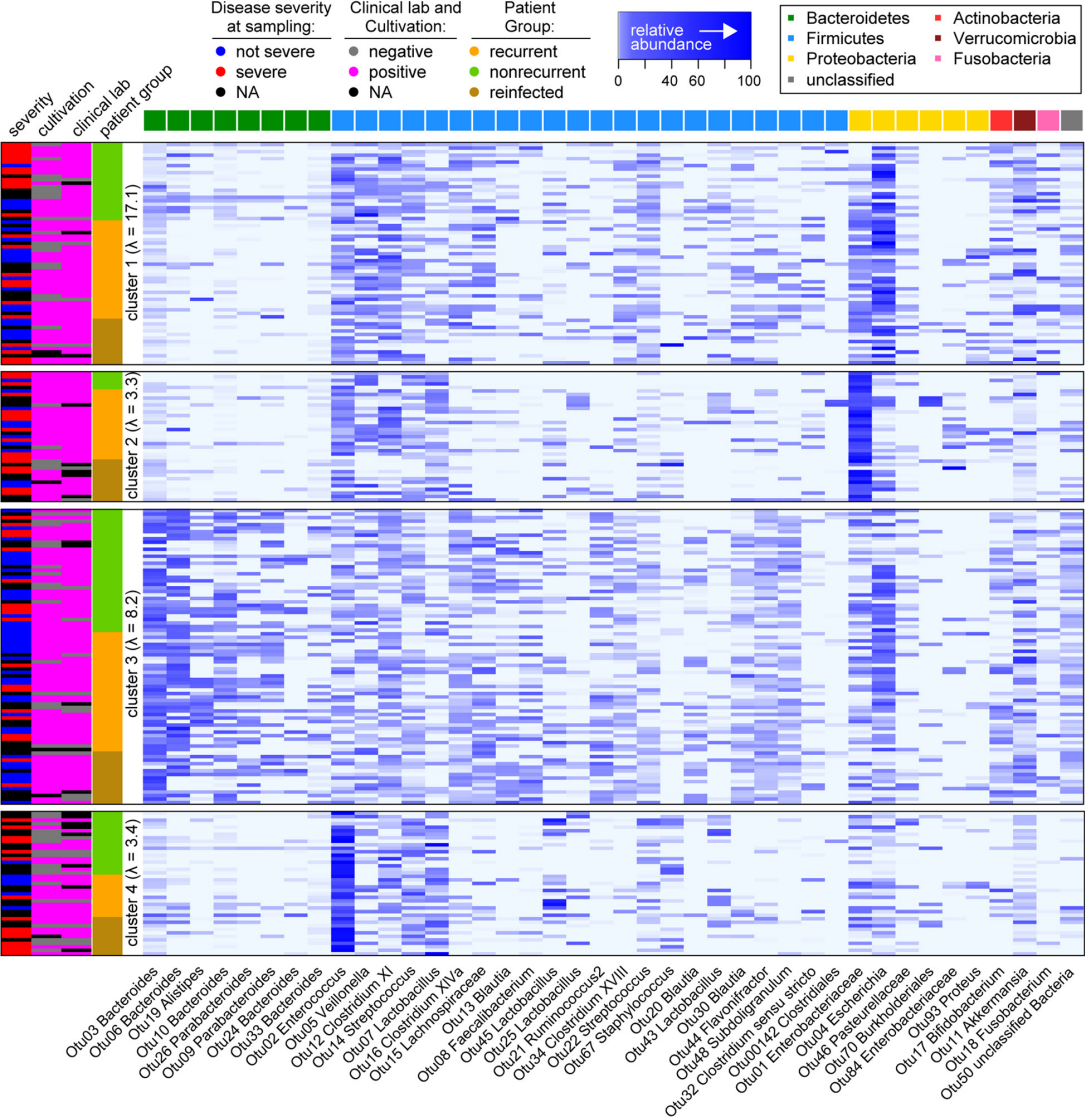
Samples clustered into four major community profiles. The relative abundance of the 40 most abundant operational taxonomic units (OTUs), with classification to the genus level and organized by bacterial phylum, is shown in columns. Samples were binned into four major clusters using the Partitioning Around Medoids (PAM) algorithm based on the Jensen–Shannon divergence. The mean inverse Simpson index (*λ*) per cluster is shown on the *left axis* (samples). Sample categorization on the *left axis* is based on the following classifications: patient group category (nonrecurrent, recurrent, or reinfected); clinical lab results (Quik Chek, positive or negative); *C. difficile* cultivation results (positive or negative); and disease severity (severe or non-severe) at sample collection during a CDI diagnosis NA = text result not available

To determine the microbial community membership and investigate shared similarities or differences within the fecal microbiota of patients with or without recurrence or reinfection, we examined the phylotypes, or genus-level taxonomic classification, of the microbial community. We employed a previously used method, Partitioning Around Medoids (PAM), clustering on the taxonomic classification of phylotypes to identify distinct community clusters [22, 29]. Investigation of the community membership in samples from all patient groups revealed variable community profiles and dominance by specific organisms as represented by OTUs in patient samples (Fig. 2). Clustering (mean *S(i)* = 0.26) of all samples resulted in four major community clusters (Fig. 2): a high-diversity cluster defined by a high relative abundance of one of two Proteobacteria members (cluster 1); a cluster of samples rich in Bacteroidetes (cluster 3); and two low-diversity clusters characterized by *Enterococcus* (cluster 4) or an Enterobacteriaceae OTU (cluster 2).

Cluster 2 contained a disproportionate amount of samples from nonrecurrent, recurrent, and reinfected samples compared with clusters 3 and 4 (Chi-square, *p* < 0.05); however, comparison of the proportion of index samples from each patient within the patient groups was not significant. When comparing the proportion of samples that were negative or positive for *C. difficile* using clinical lab results, none of the clusters was significantly disproportionate. However, when using cultivation to determine *C. difficile* status, cluster 4 contained a disproportionate amount of negative samples compared with clusters 2 and 3 (Chi-square, *p* < 0.05). Interestingly, samples during a severe diagnosis were also significantly overrepresented in cluster 4 compared with cluster 3 (Chi-square, *p* < 0.05).

**Fig. 3:**
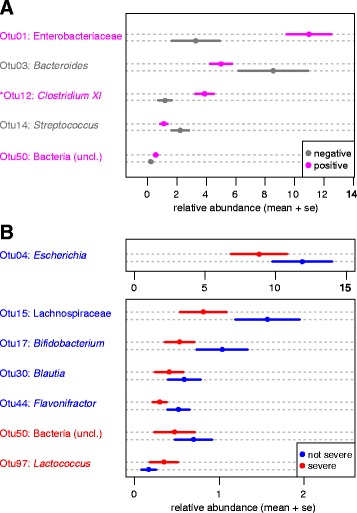
Differentially abundant members of the microbiota in patients with *C. difficile* infection. The mean relative abundance plus standard error (*se*) of differentially abundant operational taxonomic units (OTUs) identified by linear discriminant analysis (LDA) effect size (LEfSe) in (**a**) samples that tested positive or negative for *C. difficile* by the clinical laboratory (Quik Chek) or (**b**) severe or non-severe samples. OTUs that were overrepresented in the specified groups are color-coded by the respective group in each panel

In addition to clustering samples by overall community membership, we identified differentially abundant OTUs using linear discriminant analysis (LDA) effect size (LEfSe) [25]. LEfSe revealed five differentially represented OTUs in samples that were positive or negative for *C. difficile* based on University of Michigan Health System clinical lab results (*n* = 204; Fig. 3a). One of these OTUs, OTU12, classified to *Clostridium XI*, which includes *C. difficile*. While *Clostridium XI* can include other clostridial species in addition to *C. difficile*, this was the only differentially abundant OTU identified when the index (initial) samples from recurrent and nonrecurrent patients was compared using LEfSe (*n* = 93), suggesting a higher burden of *Clostridium XI*, potentially *C. difficile*, is detectable in recurrent patients at initial diagnosis (Additional file 2: Figure S1). LEfSe comparison of samples from patients with severe or non-severe disease at initial disease (using only index samples for which a severity score was available, *n* = 86) revealed seven differentially abundant OTUs with little overlap between differentially abundant OTUs between positive and negative samples (Fig. 3b). This suggests that within samples positive for *C. difficile*, patient metadata such as severity may be correlated to several different community structures.

### Microbial diversity at initial CDI diagnosis is decreased in patients with severe or recurrent disease

**Fig. 4:**
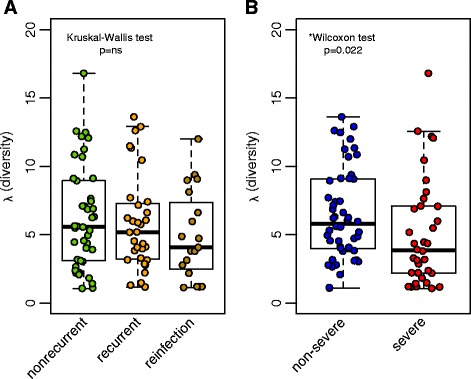
Fecal microbial diversity during initial *C. difficile* infection. The inverse Simpson index (*λ*) of the microbiota in (**a**) index samples collected at initial *C. difficile* infection (CDI) diagnosis in nonrecurrent (*n* = 42), recurrent (*n* = 32), and reinfected (*n* = 19) patients (Kruskal–Wallis, not significant (*ns*)) and (**b**) index samples from patients diagnosed with severe (*n* = 36) or non-severe (*n* = 50) CDI (Wilcoxon test, *p* = 0.022)

The diversity of the fecal microbiota community in the initial sample at diagnosis collected from nonrecurrent, recurrent, and reinfected patients was compared by calculating the inverse Simpson index (*λ*; *n* = 42, *n* = 32, *n* = 19, respectively). There was no difference in diversity when patients were classified based on clinical lab results (negative or positive for *C. difficile*), antibiotic exposure prior to initial diagnosis, or prior CDI history (Additional file 3: Figure S2). However, the fecal microbiota at initial diagnosis (index sample) in patients with recurrence trended towards a lower diversity compared with patients with nonrecurrent disease (Fig. 4a; Kruskal–Wallis test, *p* = 0.10). Furthermore, samples collected from patients with severe disease at diagnosis had lower fecal microbiota diversity compared with those without severe disease (Fig. 4b; Wilcoxon test, *p* = 0.022). Comparison of samples collected during severe or non-severe disease within the nonrecurrent, recurrent, and reinfected patient groups followed a similar trend; patients with severe CDI at the time of sample collection within each group exhibited lower diversity (Additional file 3: Figure S2).

Investigation of the microbiota diversity over time within each of the patient groups revealed time-dependent differences between patients with or without recurrence. To account for the inherent correlation present in repeated measures data, we utilized a generalized estimating equation (GEE) model to examine whether diversity (*λ*) was increased in nonrecurrent patients over time compared with recurrent patients. In this model, both time and repeated sampling are accounted for. We found that diversity and sampling across time were correlated in nonrecurrent patients, suggesting that as diversity increases across sampling time, recurrence is less likely to occur (*p* < 0.0013). In patients with recurrent disease and patients reinfected with *C. difficile,* no such increase in diversity across time was observed, suggesting that individual recovery of diversity is distinct in nonrecurrent patients compared with recurrent patients.

### The fecal microbiota community is more dynamic within patients without recurrence

**Fig. 5:**
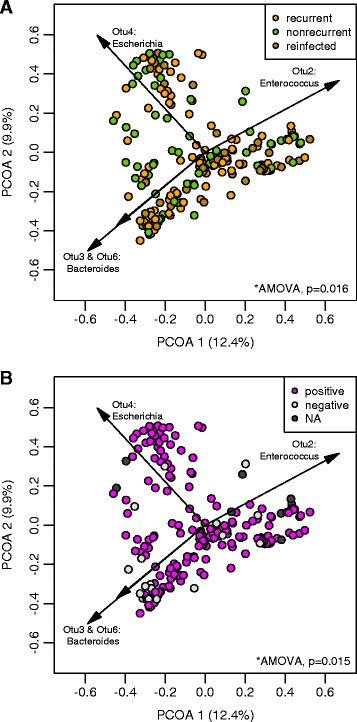
Community structure of patients with or without recurrent *C. difficile* infection. Principal coordinates analysis (PCoA) was used to plot the Yue and Clayton dissimilarity index (*θ*_*YC*_). **a** The community structure of the microbiota in samples from nonrecurrent, recurrent, and reinfected patients (analysis of molecular variance (*AMOVA*), *p* = 0.016). **b** The community structure of samples positive or negative for *C. difficile* as determined by the clinical lab using Quik Chek (*AMOVA*, *p* = 0.015)

To investigate the inter- and intra-individual similarities of the fecal community within the patients and their groups, we calculated the beta diversity using the Yue and Clayton distance (*θ*_*YC*_), a measure of similarity that accounts for abundance [23]. Principal coordinates analysis (PCoA) based on the *θ*_*YC*_ revealed significant differences between samples from recurrent, nonrecurrent, and reinfected patients using analysis of molecular variance (AMOVA) (Fig. 5a; *p* < 0.016) [26]. Comparison of the index samples (initial sample) from each patient category also trended towards being significantly different (Additional file 4: Figure S3; *p* < 0.068). We also observed significant differences between samples that tested positive or negative for *C. difficile* based on clinical results (Fig. 5b; *p* < 0.015) and using cultivation (Additional file 4: Figure S3; *p* < 0.001). A biplot of the correlating OTUs towards PCoA axes 1 and 2 revealed four OTUs responsible for opposing directions of the PCoA-determined communities: OTU4 (classified as *Escherichia*), OTU2 (classified as *Enterococcus*), and OTU3/OTU4 (both classified as *Bacteroides*) (Fig. 5).

**Fig. 6:**
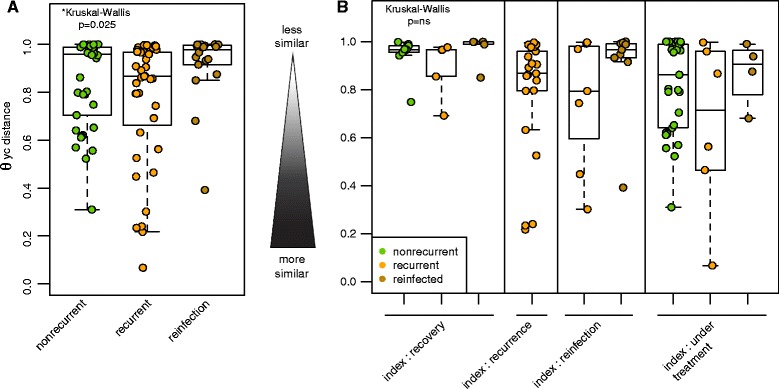
Intra-individual similarity of the microbiota in patients with or without recurrent *C. difficile* infection. The microbial community similarity within patients was compared using the Yue and Clayton dissimilarity index (*θ*_*YC*_). **a** Intra-individual similarity was lower in patients with recurrence compared with patients without recurrence or reinfected with *C. difficile* (Kruskal–Wallis test, *p* = 0.025). **b** Microbial community similarity of the index sample from a patient was compared with different stages of clinical diagnosis in nonrecurrent, recurrent, and reinfected patients: to recovery (nonrecurrent, non-reinfected samples >14 days of a positive sample), to recurrence (subsequent positive sample within 14–56 days), reinfection (subsequent positive sample >56 days), and during treatment (sample collected within 14 days of a positive sample) (Kruskal–Wallis, not significant). *ns* not significant

We next investigated how intra-individual similarity changed over time. We observed that all of the longitudinal samples collected from a given recurrent patient were more similar (*θ*_*YC*_, comparing only intra-individual samples) compared with the longitudinal samples from a nonrecurrent or reinfected patient (Fig. 6a; Kruskal–Wallis, *p* < 0.025). We compared the community dissimilarity *θ*_*YC*_ within each patient throughout consecutive sampling. Sequential comparison of changes throughout time did not reveal major differences between recurrent and nonrecurrent patients (Additional file 5: Figure S4). However, consecutive sampling does not necessarily take into account the variability in each patient’s clinical history, such as a change from index sampling (initial diagnosis) to subsequent recovery (negative for *C. difficile*) or recurrence (a second positive). To account for the variability in the clinical status of the patient throughout sampling, we annotated each sample status to reflect the patient medical history as follows: index, recurrence, reinfection, treatment, recovery (see “Methods” section for detailed definitions) (Additional file 1: Table S1). We observed greater intra-individual similarity within the recurrent group when a patient’s index sample was compared with another recurrence or reinfection, as well as recovery or treatment. Nonrecurrent patients were more likely to exhibit more dissimilarity, suggesting changes in the microbial community, during recovery and treatment phases.

## Discussion

This study represents one of the first longitudinal studies focused on the role of the microbiota in the development of recurrent CDI. Cross-sectional studies comparing the fecal microbiota of diarrheal patients with or without CDI at diagnosis with that of healthy controls have observed a range of community types, with variable community members associated with CDI [30, 31]. It is possible that incorporation of other CDI considerations, such as severity or recurrence, can impact the identification of community members that may exacerbate disease. Combined with the inherent variability already present in the human microbiota [22, 32], generalizing all patients with CDI in one group is not ideal. Indeed, studies in murine models suggest that multiple community types from different antibiotic treatments are susceptible to *C. difficile*, suggesting that disease development, and the development of recurrence, is variable and complex [33–35]. As illustrated by our data, both severity and the development of recurrence were associated with decreased diversity and community resilience. Similarly, the fecal microbiota in patients with recurrent CDI prior to fecal microbiota transplantation (FMT) has been observed to be variable, albeit severely decreased in diversity [9, 10]. The study presented here attempted to dissect some of these variables within a patient population that already does not possess a healthy microbiota.

Previous studies have used results from the clinical laboratory to determine the *C. difficile* status of a patient [30, 36, 37]. Our current study identified differentially represented OTUs as well as differential community structure between samples positive or negative for *C. difficile* based on the clinical laboratory test used at our hospital (*C. diff* Quik Chek), and cultivation of *C. difficile* from these samples generally agreed with testing results. We were unable to isolate *C. difficile* from samples during the standard antibiotic treatment window (14 days following a positive sample), potentially due to residual antibiotics in the sample. Overall, the abundance of *Clostridium XI* identified via 16S rRNA-based sequencing did not correlate with a positive test from the clinical lab or cultivation efforts. However, OTU12, belonging to *Clostridium XI*, was the only overrepresented OTU in the index sample of recurrent patients compared with nonrecurrent patients, suggesting that abundance of *C. difficile* is potentially important in the development of recurrence.

Differences in the fecal microbiota at index sampling within recurrent, nonrecurrent, and reinfected patients were less pronounced compared with differences between *C. difficile* negative or positive samples. A previous small study demonstrated that patients with recurrent disease have a less diverse community than patients with nonrecurrent CDI [38]. We did observe a slightly lower diversity in the index samples from patients with recurrence and reinfection patients compared with the index sample from patients without recurrence. However, we did find that intra-individual changes over time were more informative. Both diversity over time and overall community dissimilarity increased in patients without recurrence, suggesting that the microbial community in these patients is more dynamic. The similarity in the microbial community of samples collected during “index” and “treatment” time points in nonrecurrent patients was variable, suggesting dynamic changes in the community regardless of the test results. In contrast, there was greater similarity between samples collected during “recurrence”, “treatment”, “recovery”, or “reinfection” in patients who developed recurrence. Community types that are less susceptible to recurrence might be very individual and rely on the ability of the microbiota to change rather than the microbiota sharing a feature with that of other recurrent patients.

Surprisingly, we also observed significant differences in microbial diversity between samples collected during severe disease or not. Although LEfSe analysis revealed seven differentially present OTUs between severe and non-severe samples, few OTUs overlapped with LEfSe comparisons of *C. difficile* negative and positive samples. Comparison of patient samples categorized by severity within each of the patient groups (recurrent, nonrecurrent, or reinfected) each followed similar trends, suggesting that severity and recurrence were not associated. The severity score used in our study [12] does not reflect serious complications such as pseudomembranous colitis, ileus/toxic megacolon, or sepsis. However, it does suggest that other physiological parameters may be associated with changes in the gut microbiota and this severity score is frequently positive early in the disease process, when our index samples were collected. Given that increased gut microbiota diversity is associated with recovery from recurrent CDI following FMT, a simple severity score may be of value when deciding on treatment. Recent application of FMT for the treatment of severe disease has been effective in preventing later recurrence [39, 40]. If overall diversity of a community is in part predictive of susceptibility to recurrence, preemptive measures to promote recovery of diversity may be especially important in this patient population.

We were unable to identify a single microbiota-based metric that would predict the development of recurrent CDI. However, longitudinal analysis that considers the individual’s potential for recovery implied that patients with a more dynamic fecal microbiota were less likely to develop recurrence. This warrants analysis in a larger, more structured study to understand how recovery can be managed to decrease the likelihood of a recurrent episode and to better characterize the role of microbiota-derived variables in predictive models of severity/recurrence. As we gain a better understanding of the microbiota and their functions, which may include activities such as bile acid metabolism [35, 41], we may be in a position to identify patients at increased risk of recurrent disease and intervene through therapies that are designed to restore necessary microbiome functions.

## Conclusions

We observed distinct differences in microbiota diversity of patients with CDI that did or did not develop recurrent disease. Both static and longitudinal analysis indicated that recovery of the microbiota community is different in recurrent patients, suggesting that the overall microbiota structure may be important in susceptibility to recurrence. Additionally, disease severity at the time of diagnosis may be associated with the status of a patient’s fecal microbiota diversity. Validation of our observations in a larger cohort of patients that do or do not develop recurrence could aid in identification of microbial determinants that are associated with developing recurrent CDI.

### Ethics approval and consent to participate

All subjects signed written consent to participate in this study. This study was approved by the University of Michigan Institutional Review Board (Study HUM33286; originally approved 8/26/2009).

### Availability of data and materials

The raw sequence files supporting the conclusions of this article are available in the Sequence Read Archive (SRA) under the BioProject ID PRJNA307992, BioSamples SAMN04407535-SAMN04407764. Detailed description of data processing and generation of all figures and statistics are available at https://github.com/aseekatz/ERIN.recurrence.

## Additional files

Additional file 1: Table S1. Sample description and metadata. The relative abundance of the top 100 operational taxonomic units (OTUs), with taxonomic classification, for all samples is listed in columns. Metadata for each sample is described across each row. (XLSX 236 kb) Additional file 2: Figure S1. Relative abundance of distinct community members in patient groups. The mean relative abundance plus standard error (*se*) of operational taxonomic units (OTUs) identified by linear discriminant analysis (LDA) effect size (LEfSe) in *C. difficile*-positive and -negative samples was plotted in nonrecurrent, recurrent, and reinfected patients. LEfSe analysis identified OTU12 (*Clostridium XI*) was also overrepresented in index samples of recurrent patients compared with nonrecurrent patients. (PDF 118 kb) Additional file 3: Figure S2. Comparison of diversity in the fecal microbiota in patients with *C. difficile* infection (CDI). Comparison of the microbial diversity (inverse Simpson index *λ*) in a severe and non-severe samples in nonrecurrent, recurrent, and reinfected patients (Kruskal–Wallis test, *p* = 0.040), b *C. difficile* positive versus negative samples (not significant), c patients with or without prior antibiotic exposure (not significant) and d with or without a history of prior CDI (not significant). (PDF 173 kb) Additional file 4: Figure S3. Community structure of patients with or without recurrent *C. difficile* infection. Principal coordinates analysis (PCoA) was used to plot the Yue and Clayton dissimilarity index (*θ*_*YC*_). a The community structure of the microbiota in index (initial) samples from nonrecurrent, recurrent, and reinfected patients (analysis of molecular variance (AMOVA), *p* = 0.068). b The community structure of samples positive or negative for *C. difficile* as determined by cultivation (AMOVA, *p* = 0.001). (PDF 166 kb) Additional file 5: Figure S4. Intra-individual similarity of the microbiota in patients with or without recurrent *C. difficile* infection over sequential sampling. The microbial community similarity over sequential time within nonrecurrent and recurrent patients was compared using the Yue and Clayton dissimilarity index (*θ*_*YC*_; Wilcoxon test, not significant). (PDF 102 kb)

## Abbreviations

*θ*_*YC*_: Yue and Clayton distance
*λ*: inverse Simpson index
AMOVA: analysis of molecular variance
CDI: *Clostridium difficile* infection
ERIN: Enterics Research Investigational Network
FMT: fecal microbiota transplantation
GDH: glutamate dehydrogenase
GEE: generalized estimating equation
IDSA: Infectious Diseases Society of America
LDA: linear discriminant analysis
LEfSe: linear discriminant analysis effect size
OTU: operational taxonomic unit
PAM: Partitioning Around Medoids
PCoA: Principal coordinates analysis
PCR: polymerase chain reaction
TCCFA: taurocholate cycloserine cefoxitin fructose agar
