# Supplementary material for: Dynamics of the fecal microbiome in patients with recurrent and nonrecurrent Clostridium difficile infection
Source: Genome Med. 2016 Apr 27;8:47. doi: 10.1186/s13073-016-0298-8 (PMC4847246; doi:10.1186/s13073-016-0298-8)
Supplement: Additional file 2: Figure S1. — Relative abundance of distinct community members in patient groups. The mean relative abundance plus standard error (se) of operational taxonomic units (OTUs) identified by linear discriminant analysis (LDA) effect size (LEfSe) in C. difficile-positive and -negative samples was plotted in nonrecurrent, recurrent, and reinfected patients. LEfSe analysis identified OTU12 (Clostridium XI) was also overrepresented in index samples of recurrent patients compared with nonrecurrent patients. (PDF 118 kb) [file 13073_2016_298_MOESM2_ESM.pdf]

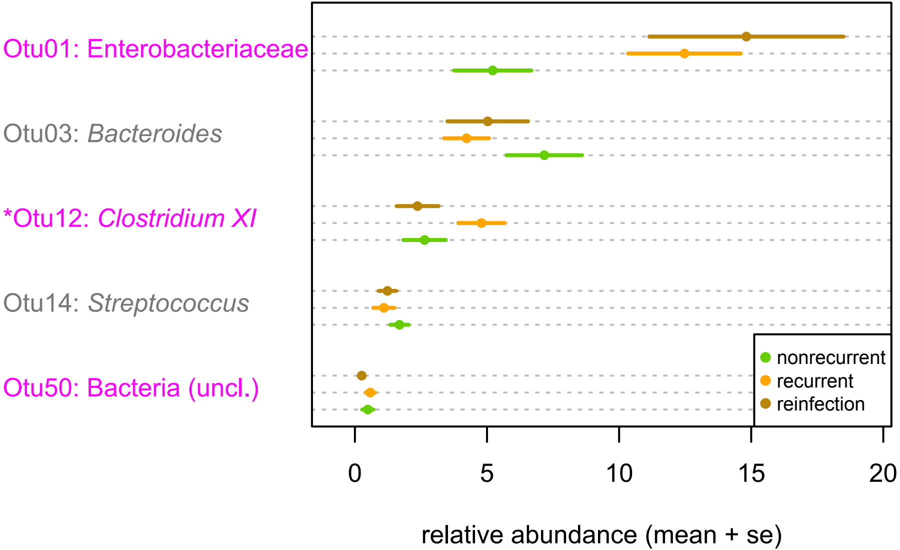
Otu01: Enterobacteriaceae
Otu03: Bacteroides
*Otu12: Clostridium XI
Otu14: Streptococcus
Otu50: Bacteria (uncl.)
nonrecurrent
recurrent
reinfection
0
5
10
15
20
relative abundance (mean + se)
