# Supplementary figures and images for: Dynamics of the fecal microbiome in patients with recurrent and nonrecurrent Clostridium difficile infection
Source: Genome Med. 2016 Apr 27;8:47. doi: 10.1186/s13073-016-0298-8 (PMC4847246; doi:10.1186/s13073-016-0298-8)

**A**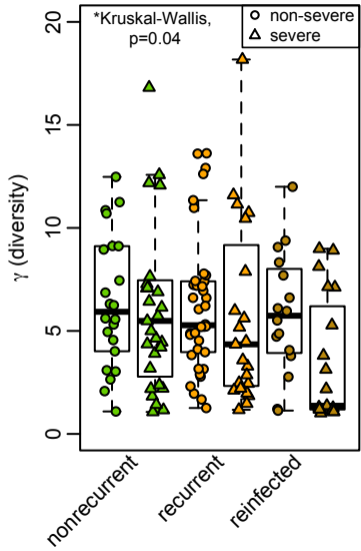**B**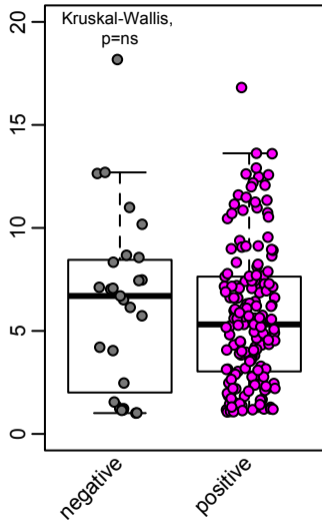**C**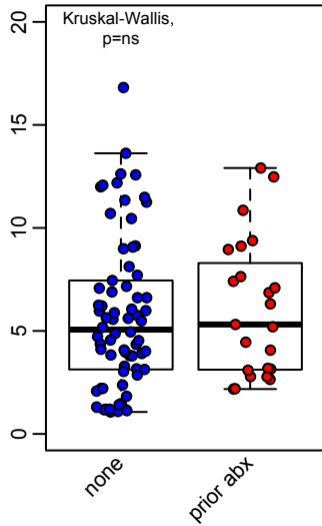**D**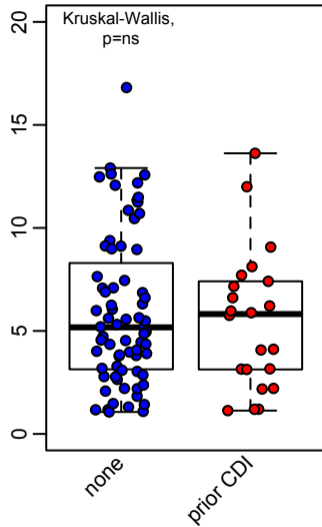

Supplement: Additional file 3: Figure S2. — Comparison of diversity in the fecal microbiota in patients with C. difficile infection (CDI). Comparison of the microbial diversity (inverse Simpson index λ) in a severe and non-severe samples in nonrecurrent, recurrent, and reinfected patients (Kruskal–Wallis test, p = 0.040), b C. difficile positive versus negative samples (not significant), c patients with or without prior antibiotic exposure (not significant) and d with or without a history of prior CDI (not significant). (PDF 173 kb) [file 13073_2016_298_MOESM3_ESM.pdf]

**A**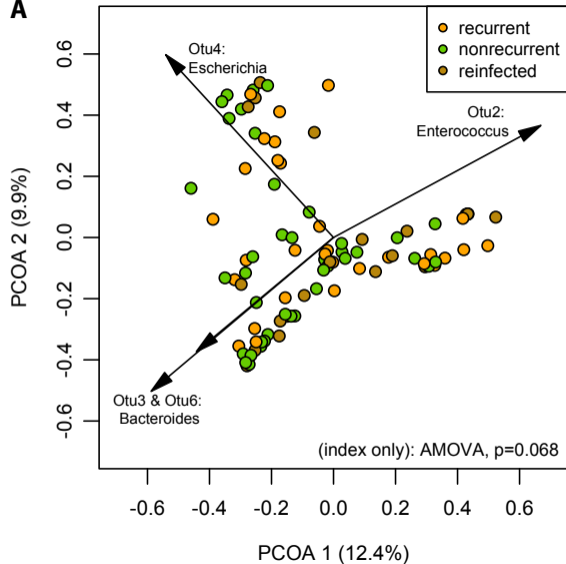**B**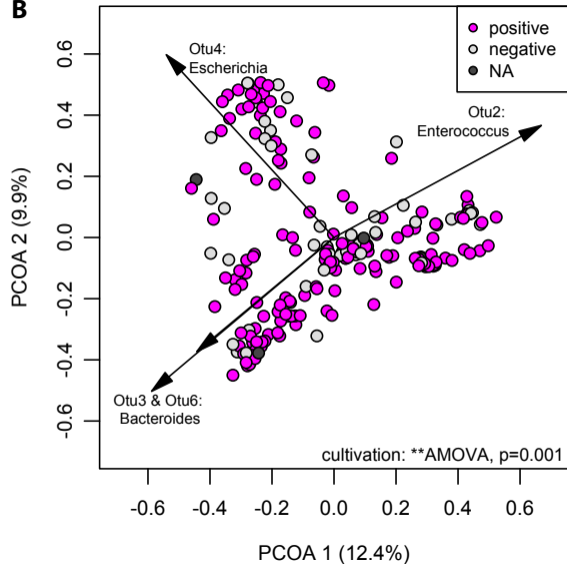

Supplement: Additional file 4: Figure S3. — Community structure of patients with or without recurrent C. difficile infection. Principal coordinates analysis (PCoA) was used to plot the Yue and Clayton dissimilarity index (θ YC). a The community structure of the microbiota in index (initial) samples from nonrecurrent, recurrent, and reinfected patients (analysis of molecular variance (AMOVA), p = 0.068). b The community structure of samples positive or negative for C. difficile as determined by cultivation (AMOVA, p = 0.001). (PDF 166 kb) [file 13073_2016_298_MOESM4_ESM.pdf]

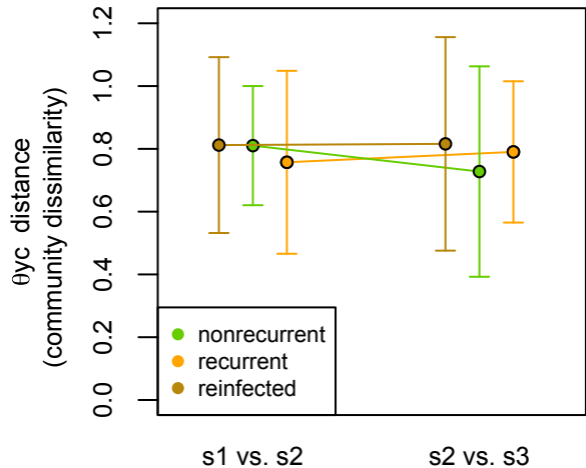

Supplement: Additional file 5: Figure S4. — Intra-individual similarity of the microbiota in patients with or without recurrent C. difficile infection over sequential sampling. The microbial community similarity over sequential time within nonrecurrent and recurrent patients was compared using the Yue and Clayton dissimilarity index (θ YC; Wilcoxon test, not significant). (PDF 102 kb) [file 13073_2016_298_MOESM5_ESM.pdf]
